# A New CT Method for Assessing 3D Movements in Lumbar Facet Joints and Vertebrae in Patients before and after TDR

**DOI:** 10.1155/2015/260703

**Published:** 2015-10-26

**Authors:** Per Svedmark, Svante Berg, Marilyn E. Noz, Gerald Q. Maguire, Michael P. Zeleznik, Lars Weidenhielm, Gunnar Nemeth, Henrik Olivecrona

**Affiliations:** ^1^Department of Molecular Medicine and Surgery, Section of Orthopaedics and Sports Medicine, Karolinska Institutet at Karolinska University Hospital Solna, 171 76 Stockholm, Sweden; ^2^Stockholm Spine Center, Löwenströmska Hospital, 198 84 Upplands Väsby, Sweden; ^3^Department of Radiology, New York University School of Medicine, New York, NY 10016, USA; ^4^School of Information and Communication Technology, KTH Royal Institute of Technology, 164 40 Kista, Sweden; ^5^School of Computing, College of Engineering, University of Utah, Salt Lake City, UT 84109, USA; ^6^Capio S:t Göran Sjukhuset, S:t Göransplan 1, 112 81 Stockholm, Sweden

## Abstract

This study describes a 3D-CT method for analyzing facet joint motion and vertebral rotation in the lumbar spine after TDR. Ten patients were examined before and then three years after surgery, each time with two CT scans: provoked flexion and provoked extension. After 3D registration, the facet joint 3D translation and segmental vertebral 3D rotation were analyzed at the operated level (L5-S1) and adjacent level (L4-L5). Pain was evaluated using VAS. The median (±SD) 3D movement in the operated level for the left facet joint was 3.2 mm (±1.9 mm) before and 3.5 mm (±1.7 mm) after surgery and for the right facet joint was 3.0 mm (±1.0 mm) before and 3.6 mm (±1.4 mm) after surgery. The median vertebral rotation in the sagittal plane at the operated level was 5.4° (±2.3°) before surgery and 6.8° (±1.7°) after surgery and in the adjacent level was 7.7° (±4.0°) before and 9.2° (±2.7°) after surgery. The median VAS was reduced from 6 (range 5–8) to 3 (range 2–8) in extension and from 4 (range 2–6) to 2 (range 1–3) in flexion.

## 1. Introduction

Common chronic low back pain (CLBP) which causes individual suffering and high societal costs [1–3] often results from painful degenerative disc disease (DDD). However, DDD is not the full explanation [4–7]. Patients often present a history of mechanical CLBP varying with different body positions, movements, and loads. The “gold standard” for treating patients failing conservative treatment is spinal fusion; however, treatment with current fusion techniques alters the biomechanics and physiological function, promoting degenerative changes in adjacent segments of the spine [8, 9]. Total disc replacement (TDR) is an alternative [10, 11].

Methods for* in vivo* analysis of TDR patients are primarily based on a two-dimensional (2D) radiographic examination where manual measurements on conventional radiographs have a precision of 2–4 mm for translation and 2–4° for rotations (depending on technique) [11–16]. Computer assisted analysis of these lateral radiographs has increased the precision to ~2° in the lumbar spine, for example, by beam distortion compensation [17, 18]. Two popular methods are distortion compensated roentgen analysis (DCRA) and quantitative motion analysis (QMA) [19]. Three-dimensional (3D) movements of a lumbar segment are provided by biplane radiography with [20] or without [21] radiostereometric analysis (RSA) and by computed tomography (CT) scans. RSA techniques with implantation of tantalum beads are an invasive method but currently the most precise method [20].

In this paper we use a noninvasive CT based method for detecting 3D movement with a radiation dose comparable to the above mentioned radiographic exam [22–24]. Four earlier studies from our group validated this method for the spine on phantom and healthy subjects and on patients with cervical total disk replacement (CTDR) and reported an accuracy of 0.6 mm and 1.0° in all cardinal planes [25–28]. This study aims to assess and compare 3D movement of individual facet joints and the segmental rotation of the vertebrae in the operated level (L5-S1) and in the adjacent level (L4-L5) in patients prior to TDR surgery and at three years after surgery.

## 2. Material and Methods

Ten patients with DDD were selected for this feasibility study (five men and five women). Median age at inclusion was 45 years (25–53). The patients meeting the criteria described below were consecutively selected from a larger randomized controlled study. Patients with a body mass index (BMI) above 35 were excluded as were patients with multilevel surgery or surgery in levels other than the L5-S1 segment. The median time of onset of back pain before surgery was 3 years (1–10). Magnetic resonance imaging (MRI) showed degeneration of the disc at the L5-S1 level with slight to moderate facet joint arthrosis. Three of these patients had done discography before surgery. Each patient signed an informed consent form which was approved by the Regional Ethics Committee (Dnr. 03-663). Three different prostheses, Prodisc (Synthes Spine, West Chester, PA, USA; *n* = 5), Charité (DePuy Spine, Raynham, MA, USA; *n* = 3), and Maverick (Medtronic, Memphis, TE, USA; *n* = 2), were used in this study.

**Figure 1:**
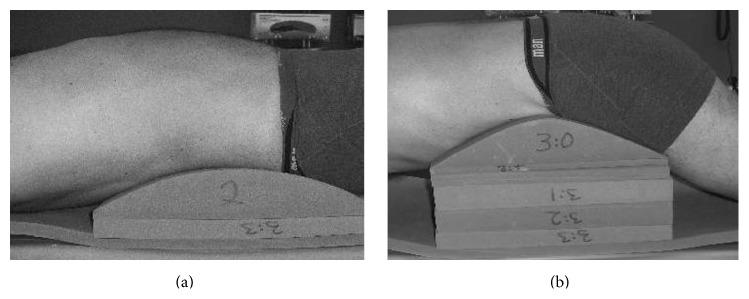
A person lying on the jig in extension provocation (a) and in flexion provocation (b).

Each patient was examined before and three years after surgery. No additional medication was given before the examinations. Each examination comprised two CT scans, one in provoked flexion and the other in provoked extension. The patients were placed on a customized jig (OT-Center, Danderyds, Sweden), incorporating different blocks for provoking the lumbar spine into extension and flexion. Provocation of the spine for extension occurred in supine position and for flexion occurred in prone position (as shown in Figure 1). Low back pain during examination was assessed using the visual analog scale (VAS) which measures pain intensity on a one-dimensional scale [29]. Patients were gradually provoked in the jig up to maximal extension or flexion but stopped if the low back pain was over 8 on the 10-level VAS scale or if in prone position the space between the top of the CT scanner tunnel and the patient's spine was too small.

The patients were examined using a clinical CT scanner (Light Speed QX/i, General Electric Medical Systems, Waukesha, WI, USA). Images were acquired from the L4 to S1 vertebra with 1.25 mm collimation, a pitch of 3 (0.75 mm/rotation), a tube tension of 120 kV, and a tube current of 250 mA. The volumes were reconstructed with an *x*-*y* matrix size of 512 × 512 into approximately 230 slices. The *x*-*y* pixel size was approximately 4 mm and the slice spacing was 0.5 mm. The radiation dose was calculated to be 0.68 mSv per scan.

Spatial registrations of CT data, and subsequent measurement of vertebrae movement, were performed using a semiautomated 3D volume registration tool described in our previous publications [25–27, 30–32]. This tool maps the “target” volume (the one to be registered) into the “reference” (the fixed) volume coordinate system. This is accomplished through selectable transformations with varying degrees of freedom, automatically generated from manually selected landmarks picked in the target and reference volumes through a graphical user interface. The registration produces two CT data volumes, the transformed target volume and the original reference volume. A more detailed technical description is given in the appendix.

**Figure 2:**
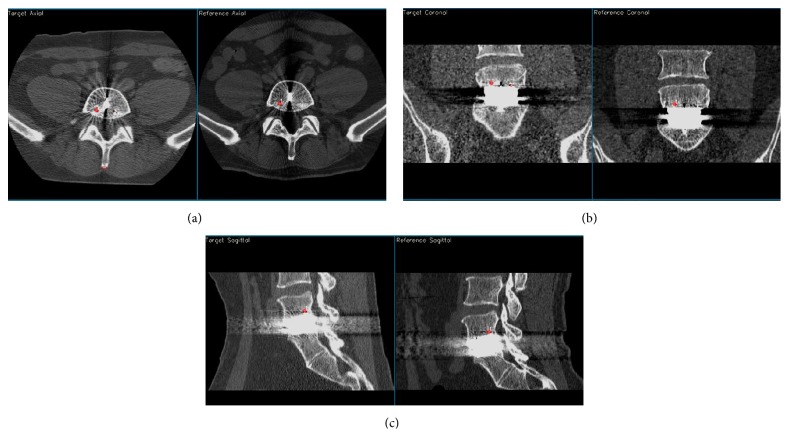
Example in 2D of choosing landmarks on L5 ((a) axial view, (b) coronal view, and (c) sagittal view).

The CT data volumes for extension and flexion were registered by using the L5 vertebra. Nine cohomologous landmarks were placed in the L5 vertebra in both CT volumes. To create an accurate registration, care was taken to spread the landmarks throughout the vertebrae in 3D, and a rigid body transformation was generated.  Figure 2 shows the placing of one landmark on L5 in all three orthogonal planes. When a landmark is chosen, the software automatically displays it on all three orthogonal planes and optionally on the 3D isosurface. Both visual and numeric analyses were used to determine how well the vertebra was registered. For the visual analysis, the registered and the reference vertebra were superimposed in 2D and in 3D as isosurfaces. For the numerical analysis the difference (in distance) between the transformed target landmarks and the reference landmarks was calculated. Ideally, this difference should be zero. In both volumes, the L5 vertebra registration was within 2 CT voxels (1.4 mm), now in a single coordinate system defined by the CT scanner with the origin at the center of the CT volume. All further rotations and translations are calculated in this “L5-registered” coordinate system. In this study the analysis was performed by one observer who was an experienced orthopaedic surgeon who was very familiar with this method. In a previous study in the cervical spine, we tested this method with regard to inter- and intraobserver difference [26].

Movement in the spine between the extension and flexion examinations was assessed by measuring (1) segmental 3D rotation and (2) facet joint 3D translation between both L4-L5 and L5-S1. These measurements utilized the 3D volume registration tool to create rigid body transformations based on sets of landmarks (as described below).

For the segmental 3D rotation, the L5-registered flexion and extension volumes were registered twice, once with respect to L4 and once with respect to S1, using landmarks placed in each of L4 and S1 exactly as was done for L5. These two registrations generated rigid body transformation matrices that corresponded to the movement between L4-L5 and L5-S1, respectively. These rotation matrices were decomposed into Euler angles to obtain the cardinal axes of the vertebra L4 and S1 in relation to L5. The following rotational order was used: *R*_*z*_ *R*_*y*_ *R*_*x*_, where *R*_*x*_ is the rotation about the *x*-axis (i.e., the sagittal plane) and this rotation was applied first.

For the facet joint 3D translation, the L5-registered flexion and extension volumes which had the individual facet joints in the same coordinate system were used. These volumes were registered twice, once with respect to L4 and once with respect to S1. To accomplish the registration, four landmarks were designated in each individual joint at the L4-L5 and L5-S1 levels. These four landmarks were placed in the two volumes as follows, for each facet joint: one each in the most cranial point, the most caudal point, the most anterior point, and a posterior point in the periphery. The volumes were then registered with respect to the L4-L5 landmarks and then again with respect to the L5-S1 landmarks. Using the translation matrix generated from the rigid body transformation the 3D translation of the left and right facet joints (as viewed from the anterior) in L4-L5 and L5-S1 was calculated. More detailed information about this method can be found in [25, 27].

The vertebral and facet joint data were tested for being a normal (Gaussian) distribution by histograms, box, density, and quantile-quantile plots. Though the data were almost normal a paired Wilcoxon signed rank test was used to evaluate the difference in motion before and after TDR. The tests for a normal distribution were applied to the pain (VAS) scores before and after TDR where data were found to be not normal. Therefore a paired Wilcoxon signed rank test was used to evaluate the difference in pain before and after TDR. The level of significant was chosen to be *p* < 0.05. The open source statistical package R version 3.0.2 was used for all statistical calculations [33].

## 3. Results

**Figure 3:**
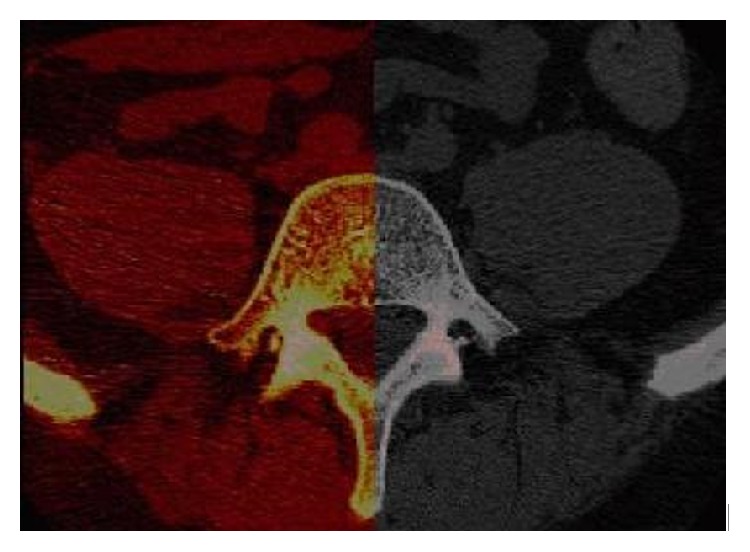
2D overlay projection of the L5 vertebra in axial plane. The red part is the transformed vertebra superimposed on the reference vertebrae. One can see that it is an almost perfect match.

All patients were able to extend and flex their spine in the jig before and three years after surgery without exceeding the pain level of 8 on the VAS scale. The space in the CT-tunnel was sufficient for maximal provocation of all patients. Patient 7 had severe pain during extension provocation both before and after surgery but it did not exceed the chosen pain level of 8 on the VAS score. Volume registration of the vertebrae was successful in all cases. In the numeric analysis, the mean value for error of all the landmarks in the human vertebrae was 0.73 mm (0.41–0.93 mm). The visual analysis of the registration of L5 is exemplified in Figure 3. With the exception of Patient 8 (see Section 4), in all other patients there were no other abnormalities nor were there any bone bridges between the vertebrae indicating spontaneous fusion.

### 3.1. Operated Level (L5-S1)

The main segmental vertebral movement was in the sagittal plane (flexion/extension), with a median (±SD) of 5.4° (±2.3°) before surgery (range 2.9–9.5°) and 6.8° (±1.7°) after surgery (range 3.8–9.6°). In all preoperative examinations, there were only small coronal and transverse plane rotations, whereas in the postoperative examinations there were some cases where the coronal rotation improved. The median (±SD) movement in the coronal plane was 0.4° (±0.2°) before (range 0.1–0.5°) and 0.8° (±1.1°) after surgery (range 0.2–3.2°) which was statistically significant (*p* = 0.03). In the transverse plane the movement was virtually unchanged. The 3D movement of the right facet joint had a median (±SD) magnitude of 3.0 mm (±1.0 mm) before (range 1.7–5.0 mm) and 3.6 mm (±1.4 mm) after surgery (range 2.6–7.6 mm). The median movement of the left joint was 3.2 mm (±1.9 mm) before (range 0.6–5.8 mm) and 3.6 mm (±1.7 mm) after surgery (range 0.3–6.7 mm). There were some asymmetric movements between the right and left facet joint both before and after surgery. Data for the individual patients are presented in Table 1. There was no significant difference found before or after surgery except in the coronal plane as already noted.

### 3.2. The Adjacent Level (L4-L5)

As in the operated level, the main movement occurred in the sagittal plane with a median (±SD) of 7.7° (±4.0°) before (range 2.2–15.2°) and 9.2° (±2.7°) after surgery (range 5.4–12.7°). In the coronal and transverse planes there were only small rotations in all patients before and after surgery. The 3D movement of the right facet joint had a median (±SD) of 3.4 mm (±2.1 mm) before (range 1.0–8.1 mm) and 3.6 mm (±1.7 mm) after surgery (range 2.5–6.9 mm). The median movement in the left facet joint was 4.0 mm (±1.9 mm) before (range 1.2–7.1 mm) and 4.5 mm (±1.8 mm) after surgery (range 2.2–7.2 mm). There were some asymmetric movements between the right and left facet joint both before and after surgery.  Table 2 presents the data for the individual patients for the adjacent L5-L4 level. There was no significant difference found before or after surgery.

### 3.3. VAS Results

Table 3 presents the VAS during the provocations of the patient. For patient 3 the pain scale was not recorded at the time of the three-year examination; therefore the pain results are based only on 9 patients. Using the Wilcoxon signed rank test, the pain during provocation in both extension and flexion three years after surgery was found to be significantly lower (*p* = 0.01 extension; *p* = 0.03 flexion) than before surgery. The median VAS in extension went from 6 before surgery (range 5–8) to 3 after surgery (range 2–8) and from 4 before surgery (range 2–6) to 2 after surgery (range 1–3) in flexion.

## 4. Discussion

This study used a method that enabled both quantitative and qualitative evaluation of motion of the vertebral segment in the lumbar spine. Detailed information was obtained about the movement of the vertebral segment, including the individual facet joint movement magnitude, before and after TDR. These patients had around 50% of the range of motion (ROM) when compared with the healthy subjects investigated previously [27]. This corresponds well with other studies contrasting the difference in mobility between healthy subjects and patients after modern TDR [18, 34]. As patients had less pain after surgery, one might expect that postoperatively patients would have a greater ROM. However, all the patients returned to almost the same ROM after surgery, and considering the large difference in ROM between the patients preoperatively, preoperative ROM might be the most important factor in predicting postoperative ROM [35]. A lateral view (2D X-ray) only presents one cardinal axis. Even though there are some methods to calculate the other two axes, it is not a truly three-dimensional method. This method is a true three-dimensional method and we know that the segmental movement is a three-dimensional movement and not just a 2-dimensional movement in the sagittal plane. Additionally, we did not note any ossification between the endplates of the vertebrae in the operating level or at the adjacent levels in the follow-up CT scans after three years. No major complication had occurred to any of the patients after three years.

This evaluation was based upon passive provocation; thus, the muscle contraction was less in the lumbar spine with this provocation than it would be if the patients were provoked in a standing position. Some studies have shown that passive provocation has an increased ROM compared to active muscle provocation [13]. However, with passive provocation the motion of the facet joints might better reflect the relationship between implant and facet joint than the facet joints' actual motion pattern. In our provocations, we encountered two practical problems. First, as the duration of patient provocation increased, the risk of motion artefacts increased. However, with present day CT scanners this is much less of an issue. Second, prone flexion provocation can be limited by the CT-tunnel (although this did not occur in this study). It was important to place the jig in the correct position in relationship to the pelvis so that maximum provocation in both extension and flexion would be in the lower lumbar spine. Extension provocation was easy to perform in this study, with all patients able to be fully extended.

The radiation exposure from CT examinations is, in general, higher than that from conventional radiographic examinations. However, with modern CT scanners the protocol can be optimized to reduce the effective radiation dose. In this study the CT scan protocol was adjusted to reduce the effective radiation dose to as low as 0.68 mSv/CT scan, which is almost equal to that of conventional 2D radiographic examination [36, 37]. Future development of CT scanners will further reduce the amount of radiation and thus increase the possibility to improve the accuracy of this method by allowing higher resolution with the same radiation exposure. Additionally, metal artefacts have been decreasing in CT scans because the manufacturer's software has been increasingly handling them better.

Proponents argue that TDR preserves/restores segmental motion and might reduce the risk of adjacent segment degeneration (ASD). Some studies support the argument that ASD is prevented after TDR [38–40], while others show ASD after TDR only with the first type of disc prosthesis that was used [41, 42]. This could be the result of the DDD progressing by itself to multiple levels; it might be a consequence of increased stress on adjacent levels generated from nonphysiological motion, or lack of motion in the disc prosthesis [41].

Prostheses that fail to adequately replicate the physiologic kinematics of the lumbar spine may predispose the patient to facet joint degeneration at the treated segment or in adjacent segments. The distance and angle after TDR are reproducibly obtained with CT after surgery. We have done a number of studies with double examinations where this is shown to be true. For instance, see [26]. Therefore, we believe that it is important to analyze if any changes occur in the individual mobility pattern of facet joints at both operated and adjacent segments after TDR [43]. Analysis of 3D movement and evaluation of the individual facet joints before and after surgery can reveal how the disc prosthesis affects the motion pattern of the segment.

The present study was a small study with only ten patients in which the same experienced orthopaedic surgeon inserted all the prosthesis. The intended position of the implant was all the way towards the posterior longitudinal ligament, since that would place the center of rotation in the physiological anterior-posterior position. To avoid irregular mobility and load that could over time affect either facet joint, the implant was positioned as close to the midline as possible. When this study was undertaken, implants were higher than today, which is why sometimes the treated segment might have been “overstuffed,” but in general the lowest implants available were used [44]. Further, X-rays were taken shortly after surgery, which showed that the prosthesis was correctly aimed and positioned with regard to the size and height of the devise. The aim was to establish the possibility to detect rotation and facet joint movement. In this regard it was interesting to look at the quality of motion, as in the following examples.

**Figure 4:**
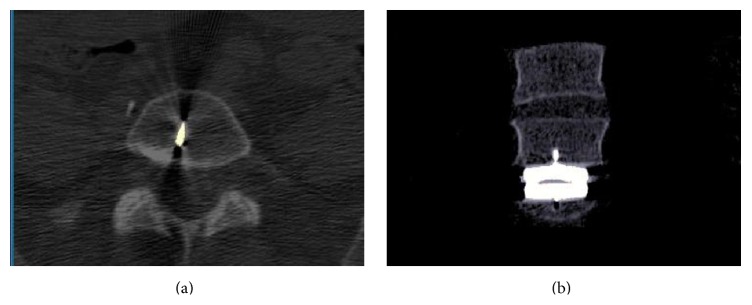
Patient 1: 2D projections where the prosthesis is visualized as towards the right of the midline. In the left axial view, the keel of the prosthesis is partly visualized. In the right coronal view it is shown that the prosthesis was placed slightly to the right of the midline.

Patient 1 had a Maverick prosthesis in the L5-S1 level. The rotation after surgery was about the same as before surgery, but the magnitude of the facet joint movement was different: the movement in the left joint was 6.7 mm and in the right facet joint was 3.5 mm. Visual analysis showed that the prosthesis was placed slightly to the right of the midline of the vertebra (Figure 4).

**Figure 5:**
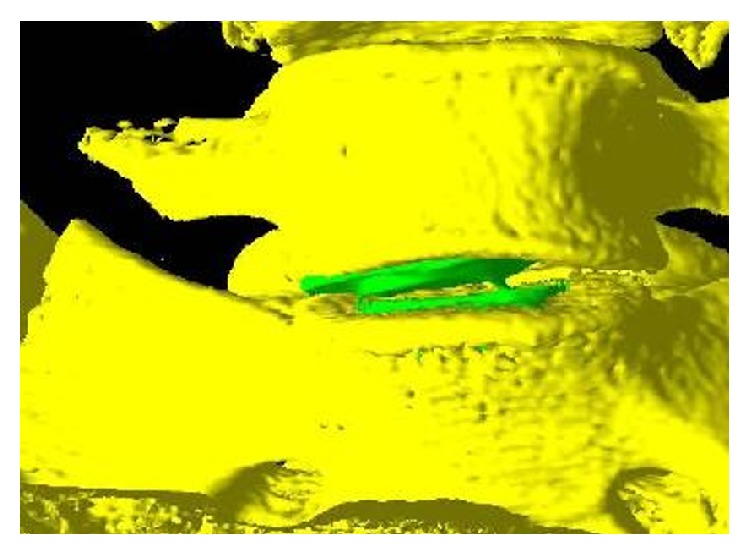
3D isosurface illustrating the subsidence of the prosthesis (green) into the endplate of the L5 vertebra on Patient 8.

In Patient 8, the Charité prosthesis had subsided into the L5 vertebra and caused a coronal plane rotation and a different movement in the facets joints (Figure 5). The asymmetric facet joint movement might indicate that the facet joint with more stress might degenerate sooner with the possible recurrence of low back pain.

**Figure 6:**
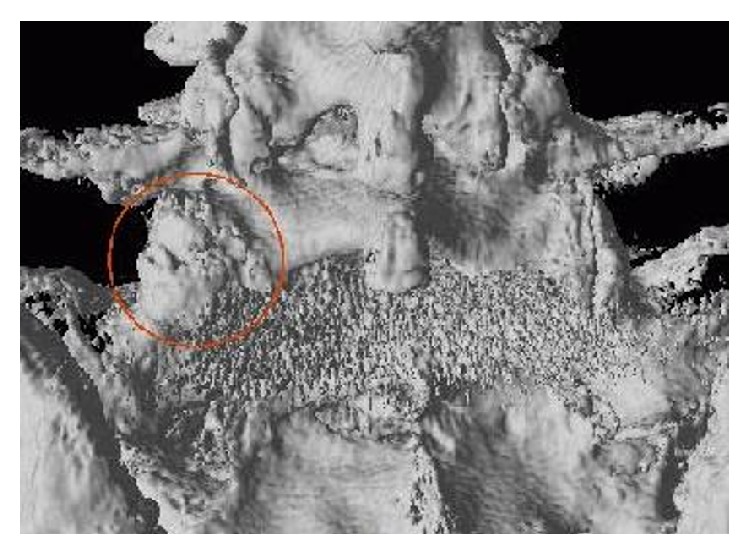
3D isosurface for Patient 4 viewed from behind the L5-S1 vertebrae. In the left joint of L5-S1 (red circle) the osteoarthritis is severe with visually displayed osteophytes in this view. Below the joint there are artefacts from the prosthesis in L5-S1 level.

In Patient 4 the left facet joint was more degenerated with osteophytes in the CT scans three years after surgery than the right facet joint, and it clearly induced a large difference in the coronal rotation in addition to there being a large asymmetry between the movement of the left and right facet joint (Figure 6).

This method is a truly 3D method that is easy to use, is noninvasive, and can be performed routinely in a clinical setting using any modern CT scanner, with an effective radiation dose comparable to radiographs. It takes five to ten minutes to complete the registration of the L5 vertebra. It then takes another five to ten minutes to choose landmarks on the L4 and S1 vertebrae and a comparable time for landmark choice on the facet joints. Then the analysis proceeds automatically and completes in under a minute. The data found in this small pilot study suggests that TDR preserves the motion in the lumbar segment even after three years which might cause less adjacent level problems.

## 5. Conclusion

It has been shown that this method is suitable to study patients operated on in the lumbar spine. This truly 3D method can be performed in a relatively short time at low effective radiation dose. Detailed information about kinematics was obtained. This method of detecting movement in the spine is useful in research to confirm the correct positioning of disc prostheses, in the future development of TDR designs, and for clinical use.

## Figures and Tables

**Table 1:** Individual patient's movement and facet joint magnitude at L5-S1 level before and after surgery.

	Rotation (degrees)						3D facet translation (mm)			
Patient	Sagittal		Coronal^†^		Transverse		Right		Left	
	Before	After	Before	After	Before	After	Before	After	Before	After
1	9.3	9.6	0.4	0.7	0.0	0.9	4.8	3.5	5.7	6.7
2	9.5	8.6	0.5	0.5	0.1	1.0	5.0	7.6	5.0	3.7
3	3.7	3.8	0.1	0.2	0.4	0.0	2.5	2.6	1.7	1.7
4	5.5	5.3	0.5	3.2	0.9	0.5	3.7	3.0	1.4	0.3
5	8.2	5.9	0.1	0.8	0.1	0.2	3.8	3.5	4.1	3.4
6	4.3	7.4	0.5	1.9	0.5	0.6	1.7	4.0	5.8	3.1
7	2.9	6.6	0.2	0.8	0.1	0.6	2.7	4.0	0.6	4.4
8	6.1	6.9	0.2	2.7	0.6	0.3	3.1	2.6	1.9	4.1
9	4.3	5.0	0.5	0.2	0.8	0.4	2.7	3.7	4.7	4.6
10	5.3	7.4	0.4	0.4	0.8	0.0	2.8	3.6	2.3	2.5

Median (±SD)	5.4 (±2.3)	6.8 (±1.7)	0.4 (±0.2)	0.8 (±1.1)	0.5 (±0.3)	0.5 (±0.3)	3.0 (±1.0)	3.5 (±1.4)	3.2 (±1.9)	3.7 (±1.7)

Minimum	2.9	3.8	0.1	0.2	0.0	0.0	1.7	2.6	0.6	0.3

Maximum	9.5	9.6	0.5	3.2	0.9	1.0	5.0	7.6	5.8	6.7

^†^Significantly different before and after operation (*p* = 0.03).

**Table 2:** Individual patient's movement and facet joint magnitude at L4-L5 level *before* and *after* surgery.

	Rotation (degrees)						3D facet translation (mm)			
Patient	Sagittal		Coronal		Transverse		Right		Left	
	Before	After	Before	After	Before	After	Before	After	Before	After
1	7.1	6.8	0.3	1.0	0.3	0.2	3.4	2.6	3.3	2.2
2	11.6	12.7	1.4	0.8	0.0	0.3	3.2	3.7	4.5	5.8
3	2.2	9.6	0.8	0.8	0.4	0.4	1.0	5.6	1.2	6.6
4	3.8	6.5	1.1	0.4	0.2	0.3	1.9	3.4	2.0	3.8
5	15.2	12.4	0.2	1.1	0.7	0.4	8.1	6.9	7.1	6.7
6	5.9	8.8	0.6	0.5	0.9	0.9	2.8	3.2	3.1	3.9
7	3.2	5.4	0.6	1.4	0.8	0.9	3.4	2.5	3.4	2.7
8	9.8	8.7	0.2	2.2	1.1	1.3	4.6	2.9	6.1	3.0
9	9.2	12.7	0.8	1	0.8	0.4	3.9	4.9	4.6	5.1
10	8.2	10.3	1.2	0.9	0.2	0.3	6.2	6.7	5.9	7.2

Median (±SD)	7.7 (±4.0)	9.2 (±2.7)	0.7 (±0.4)	1.0 (±0.5)	0.6 (±0.4)	0.4 (±0.4)	3.4 (±2.1)	3.6 (±1.7)	4.0 (±1.9)	4.5 (±1.8)

Minimum	2.2	5.4	0.2	0.4	0.0	0.2	1.0	2.5	1.2	2.2

Maximum	15.2	12.7	1.4	2.2	1.1	1.3	8.1	6.9	7.1	7.2

**Table 3:** The visual analogue scale (VAS) for pain before and three years after surgery during provocation in extension and flexion. Patient number three was excluded because there were no scores at three-year examination.

Patient	VAS extension^†^		VAS flexion^†^	
	Before	After	Before	After
1	5	2	3	2
2	6	5	4	3
3	Missing value	Missing value	Missing value	Missing value
4	6	3	4	2
5	6	3	3	3
6	7	3	6	3
7	8	8	5	3
8	5	2	2	2
9	7	4	2	2
10	6	3	4	1

Median	6	3	4	2

Minimum	5	2	2	1

Maximum	8	8	6	3

*p* value		0.01		0.03

^†^Significantly different before and after surgery.

## Appendix

### Technical Note

The user interface of the volume registration tool can simultaneously present, for any pair of volumes, arbitrary 2D slices from any orthogonal plane (axial, sagittal, and coronal) as well as 3D isosurfaces. The slices can be viewed in two larger display windows representing one orthogonal plane for each volume or six smaller windows representing all three orthogonal planes for each volume. A number of color, black and white, and gray scales are available. For CT volume viewing, one or two window width/level settings can be used as desired. For example, a lower window can be used for viewing skeletal structure or soft tissue, while a higher window can be used for simultaneously viewing metal or other high attenuating structures. The 3D isosurfaces can be viewed in large or small formats and can be zoomed, rotated, and viewed from any arbitrary direction, with various surface types (e.g., opaque or translucent).

Landmarks are chosen on concurrently viewed slices or directly on 3D isosurfaces which display the same physiological structure and are recorded as true 3D coordinates (automatically mapped to closest slices for display). Landmarks can be chosen as simple 3D points or with the aid of a 3D sphere. The sphere landmark superimposes contours of a 3D sphere that, when designated, returns the 3D coordinates of the sphere's center as the landmark point. When a landmark is chosen, the view of the slices is updated in all three orthogonal planes, the corresponding point in the 3D volume is recorded in units of mm (rather than voxel coordinates), and a sequence number is generated.

The 3D paired landmarks are used to generate the eigenvalues of the transformation matrix of coefficients. For nonrigid registration the eigenvalues are generated by employing a weighted least square linear regression followed by a Gauss-Jordan matrix inversion. For rigid body registration singular value decomposition is employed. Either method limits the effect of mismatched landmarks and generates transform coefficients for arbitrary volume data sets. At present, the *x*, *y*, and *z*-coordinates are given equal weight in the transformation, although there normally is a finer resolution in the *x* and *y* directions when compared with the *z* direction. It is possible to weight the linear regression or the singular value decomposition to compensate for this difference, but presently this is not done. There is also the possibility to use a completely manual affine transformation. The tool also offers a nonaffine transformation in the form of a first- and second-degree transformation which does not preserve the original voxel coordinate grid. Finally, a transformation is performed.

Once the new transformed volume has been created from the original, untransformed data set, this transformed volume may be resliced and evaluated side by side with the original volume in any of the three planes or superimposed on the original slices. An isosurface of the transformed volume may be displayed in 3D, superimposed with an isosurface from the original volume and/or the untransformed volume for comparison. Landmarks can also be utilized with the transformed volume together with the original volume either to create a better match or to generate a numeric estimate of the registration error.

The application in the present study used a rigid body transform. This type of transformation preserves the spatial integrity of the structures involved. Landmarks were chosen on the registered transformed and original volumes to generate numeric values for the correlation of the L4 and S1 vertebral rotations and facet joint translational movement.
